# Human liver CEST imaging at 7 T: Impact of $B_{1}^{+}$ shimming

**DOI:** 10.1002/mrm.30557

**Published:** 2025-05-24

**Authors:** Petr Bulanov, Petr Menshchikov, Johannes A. Grimm, Max Lutz, Stephan Orzada, Philip S. Boyd, Peter Bachert, Mark E. Ladd, Andreas Korzowski, Sebastian Schmitter

**Affiliations:** ^1^ Division of Medical Physics in Radiology German Cancer Research Center (DKFZ) Heidelberg Germany; ^2^ Faculty of Physics and Astronomy Heidelberg University Heidelberg Germany; ^3^ Faculty of Medicine Heidelberg University Heidelberg Germany; ^4^ Medical Physics and Metrological Information Technology Physikalisch‐Technische Bundesanstalt (PTB) Berlin Germany; ^5^ Center for Magnetic Resonance Research University of Minnesota Minneapolis Minnesota USA

**Keywords:** 7 T, B_1_ correction, B_1_+$B_{1}^{+}$ shimming, body CEST, liver CEST, pTx

## Abstract

**Purpose:**

To explore the feasibility of CEST imaging in the human liver at 7 T with $B_{1}^{+}$ shimming.

**Methods:**

CEST MRI was performed on a 7 T whole‐body scanner with a parallel transmission (pTx) system in five healthy volunteers. Static pTx ($B_{1}^{+}$ shimming) was applied to locally maximize the $B_{1}^{+}$ magnitude per input power within a given region of interest (ROI) of approximately 30 mm diameter (ROI_shim_). Relaxation‐compensated inverse magnetization transfer ratio (MTR_Rex_) values were quantified for amide protons, guanidino protons, and relayed nuclear Overhauser effect signals based on five‐pool Lorentzian fit analysis. MTR_Rex_ values were corrected for B_1_ inhomogeneities using an absolute, accurate MR fingerprinting–based $B_{1}^{+}$ mapping technique.

**Results:**

Within the ROI_shim_, reliable MTR_Rex_ values could be calculated for an average of 85% of voxels. The mean MTR_Rex_ values and corresponding coefficient of variations across the group are: 0.113 ± 0.009, 8.8% for amide; 0.167 ± 0.010, 6.3% for nuclear Overhauser effect; and 0.079 ± 0.010, 12.9% for guanidino. MTR_Rex_ values exhibit low variation between subjects, as reflected by low coefficient of variations.

**Conclusion:**

In this study, we have demonstrated for the first time the feasibility of acquiring and quantifying relaxation‐compensated CEST contrasts in the human liver at ultrahigh field. The application of static pTx effectively eliminates $B_{1}^{+}$ dropouts and allows for accurate CEST contrast quantification within the selected ROI. In addition, the proposed $B_{1}^{+}$ mapping technique shows efficacy for enhanced MTR_Rex_ B_1_ corrections in the abdomen.

## 1 INTRODUCTION

CEST imaging is a promising technique that provides a unique MRI contrast mechanism. It reflects the indirect detection of molecules that contain exchangeable protons.^1^, ^2^, ^3^ CEST imaging is increasingly employed as a valuable quantitative biomarker, especially for the characterization of brain tumors.^4^ In particular, it is utilized for the identification and grading of tumors^5^, ^6^, ^7^, ^8^ and the evaluation of treatment efficacy.^9^, ^10^, ^11^, ^12^, ^13^, ^14^, ^15^, ^16^

Whereas most studies focus on the brain, CEST imaging outside the brain shows promise, although it is less widespread and more prone to artifacts, and therefore requires further investigation.^17^ Liver CEST imaging, in particular, might be promising for amide proton transfer‐weighted imaging,^18^, ^19^, ^20^ glycogen imaging,^19^, ^21^, ^22^ and extracellular pH measurements in human benign and malignant liver tumors.^23^ Compared to the brain, however, CEST imaging of the body faces several technical challenges,^24^ including motion artifacts, strong B_0_/B_1_ inhomogeneities, power and specific absorption rate restrictions, fat signal interference, and a more heterogeneous tissue composition. Some of the difficulties are especially significant for liver imaging because it is one of the largest solid internal organs in the human body. Likely due to these reasons, thus far there is only a small number of CEST studies targeting the human liver.^20^, ^25^, ^26^, ^27^, ^28^ Furthermore, all of these studies were performed at 3 T. Additionally, due to insufficient spectral resolution and SNR, magnetization transfer ratio (MTR) asymmetry^29^ analysis was performed, which is less informative than the relaxation‐compensated inverse metric, that is, the MRT of the exchange‐dependent relaxation rate in the rotating frame (MTR_Rex_).^2^, ^30^ Thus, higher SNR and spectral resolution are desirable for pursuing liver CEST using MTR_Rex_.

Performing CEST imaging at 7 T would address the SNR problem and would have the significant benefit^31^, ^32^ of providing a higher spectral resolution, which is expected to result in a better separation of the different CEST signals appearing in the Z‐spectrum. Better spectral separation in the Z‐spectrum allows for more accurate extraction of each signal and, consequently, calculation of MTR_Rex_ values.^33^ However, abdominal CEST MRI at ultrahigh field is challenged by noticeable B_0_/B_1_ inhomogeneities and, particularly, by the presence of $B_{1}^{+}$ dropouts when the coil elements have non‐optimized phase settings, for example, identical phase applied across all transmitter channels. Furthermore, in the abdominal area $B_{1}^{+}$ dropouts cannot be corrected with traditional circularly polarized (CP) transmission in which the phase of each transmitter channel is shifted by 360°N, where N is the number of transmitter channels and assuming the elements are spaced equally, allowing their $B_{1}^{+}$ fields to coherently combine. Accordingly, the CP transmission results in $B_{1}^{+}$ field combination at the center of the object, which is highly effective in brain imaging. However, in the abdomen, it leads to off‐center $B_{1}^{+}$ dropouts, which may overlap with the target region in the liver.

A 2D gradient‐echo (GRE) CEST sequence^34^ includes both saturation and readout pulses, each applied with different nominal flip angle (FA) values and influenced by the $B_{1}^{+}$ inhomogeneity. $B_{1}^{+}$ dropouts arise from insufficient FA, substantially reducing the efficacy of CEST saturation and yielding low SNR due to both low saturation and readout FA. Consequently, mitigating $B_{1}^{+}$ dropouts is a key prerequisite for body CEST imaging at 7 T.

However, even if no complete $B_{1}^{+}$ dropouts are present in the target region, the spatial inhomogeneity of $B_{1}^{+}$ and consequent variations in FA of the saturation pulses also can cause nonuniform saturation efficiency across the imaging volume. This affects not only the SNR of the CEST signals but also the contrast in the CEST images.^30^, ^35^ Each CEST signal (amide, guanidino, exchange‐relayed nuclear Overhauser effect [rNOE], and semi‐solid magnetization transfer [ssMT]), as well as direct water saturation (‘“spillover”’), strongly depend on $B_{1}^{+}$ and reach a maximum contribution at specific $B_{1}^{+}$ values.^35^, ^36^, ^37^

To overcome the $B_{1}^{+}$ dropouts and $B_{1}^{+}$ variations in the human liver at 7 T, static parallel transmission (pTx) techniques, also termed $B_{1}^{+}$ *shimming*,^38^ have been successfully applied.^39^ The static pTx approach, which can be combined in a straightforward manner with acquisitions such as CEST, is employed to achieve a subject‐specific optimization of the resulting superimposed spatial $B_{1}^{+}$ pattern within a region of interest (ROI), which directly translates into an optimized FA pattern. The desired $B_{1}^{+}$ pattern is created by using a multichannel transmit coil array and applying channel‐specific complex scaling factors to the RF pulses to generate the intended superimposed spatial $B_{1}^{+}$ field.

In the current study, we address $B_{1}^{+}$ dropouts and $B_{1}^{+}$ variations and explore the feasibility of CEST imaging in the liver in healthy volunteers at 7 T. To this end, we demonstrate (i) the need and effectiveness of applying static pTx to overcome dropouts of $B_{1}^{+}$ in the target region in order to enable appropriate CEST contrast preparation, and (ii) the acquisition of accurate $B_{1}^{+}$ maps enabling the B_1_ correction of individual CEST contrasts. With this, to the best of our knowledge, we demonstrate for the first time the robust acquisition of MTR_Rex_ CEST contrasts, that is, amide, guanidino, and rNOE MTR_Rex_ maps of the human liver at ultrahigh field.

## 2 METHODS

### 2.1 MR acquisition

MR examinations were performed using a 7 T whole‐body MR scanner (Magnetom 7 T, Siemens Healthineers, Erlangen, Germany) equipped with a pTx system with a local eight‐channel transmit/receive body coil.^40^ CEST imaging on the pTx system was validated using a bovine serum albumin phantom prior to the in vivo study (c.f. Figure S1 and Figure S2).

**FIGURE 1:**
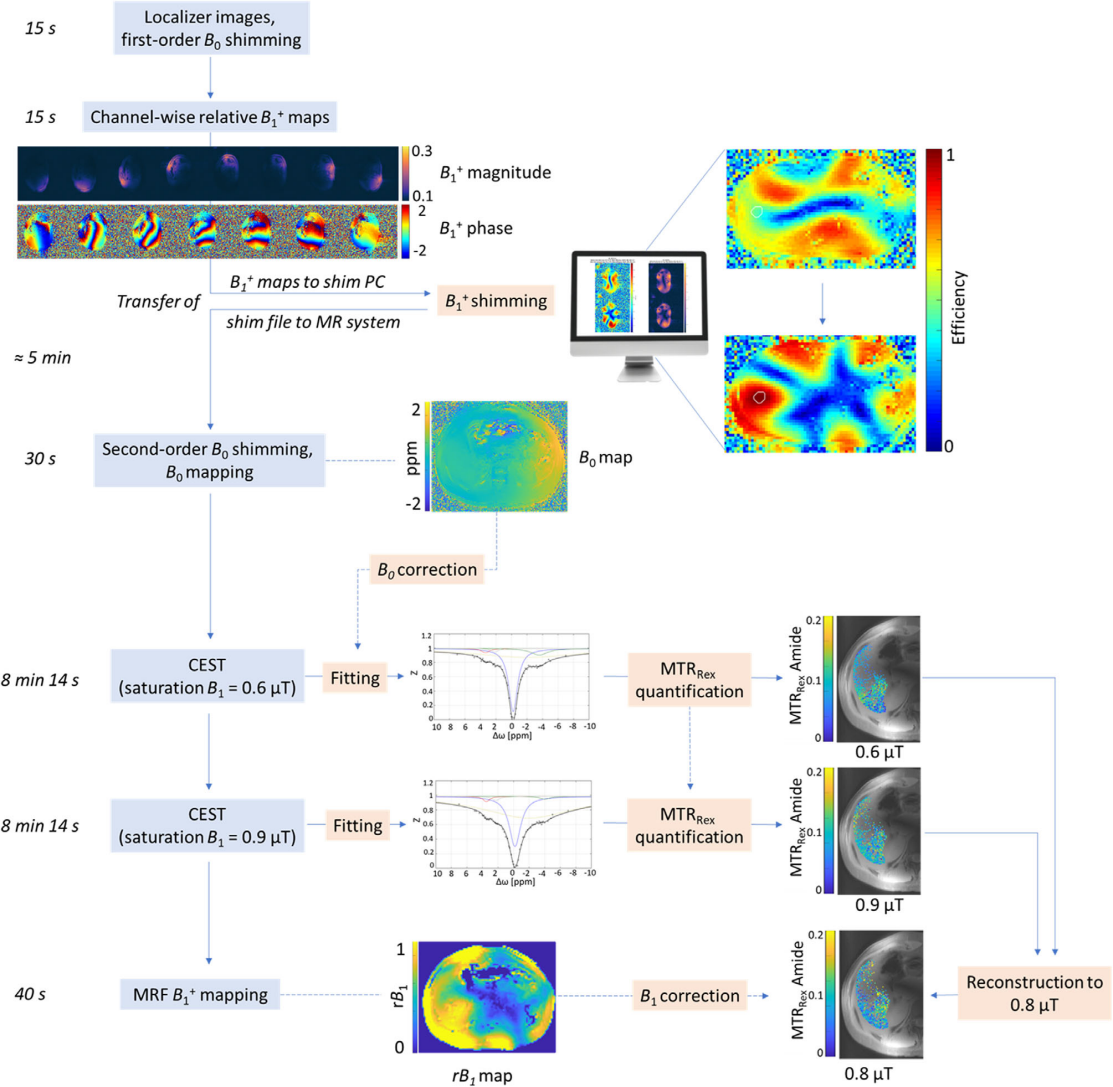
Flowchart of the acquisition (blue boxes) and postprocessing steps (orange boxes). The MRI protocol included localizer imaging and first‐order B_0_ shimming, followed by channel‐wise relative $B_{1}^{+}$ mapping. $B_{1}^{+}$ shimming was then performed on a stand‐alone computer, which required transferring the channel‐wise $B_{1}^{+}$ maps to this computer and then returning the optimized shim file to the MR system. Afterward, second‐order B_0_ shimming and B_0_ mapping were conducted. CEST acquisitions were then performed with two nominal saturation B_1_ values: 0.6 and 0.9 μT. For CEST B_1_ correction, free‐breathing MRF‐based $B_{1}^{+}$ mapping was employed. MRF, MR fingerprinting.

**FIGURE 2:**
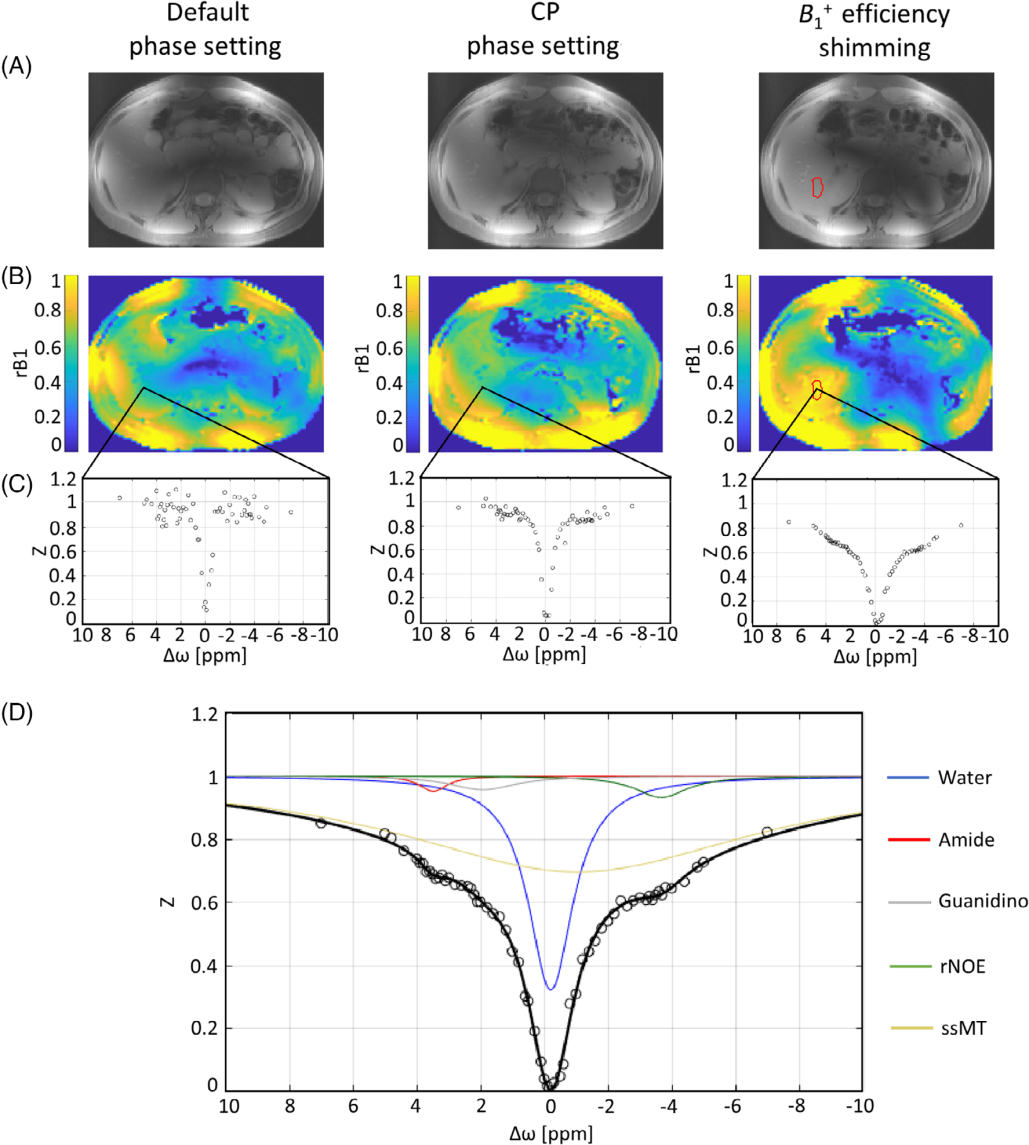
Structural images (CEST *M*_0_ at Δω = 300 ppm) (A) and *r*B_1_ maps (B) specified for the different transmission phase settings: default (left column), CP (middle column), and the optimized static phase setting ($B_{1}^{+}$ efficiency shimming, right column). The ROI (ROI_shim_) used for the $B_{1}^{+}$ shimming procedure is indicated by the red line. (C) Representative Z‐spectra for each phase setting obtained from the same voxel within ROI_shim_ (nominal saturation amplitudes B_1 nom_ = 0.9 μT, *r*B_1_ = 0.49, 0.59, and 0.95 for default, CP phase, settings and $B_{1}^{+}$ efficiency shimming, respectively). $B_{1}^{+}$ shimming leads to an increase in the Z‐spectrum quality inside the target region, which corresponds to higher *r*B_1_. (D) Example of five‐pool (water, amide, guanidino, rNOE, and ssMT) Lorentzian model fitting of the representative Z‐spectrum obtained with $B_{1}^{+}$ efficiency shimming. CP, circular polarization; rNOE, relayed nuclear Overhauser effect; *r*B_1_, relative B_1_ values; ROI, region of interest; ssMT, semisolid magnetization transfer.

All subject measurements were performed after a 10‐h fasting period. A schematic flowchart of the MRI acquisition protocol (total duration: 23 min) and postprocessing steps is shown in Figure 1. After the acquisition of localizer images, first‐order B_0_ shimming was performed with the following parameters: GRE, resolution: 4 × 4 × 4 mm^3^; TE1 = 2.04 ms; TE2 = 4.08 ms; TR = 367.8 ms; nominal FA = 15°; readout bandwidth (BW) 651 Hz/px; acquisition time (TA) = 4 s. For static parallel transmission (i.e., $B_{1}^{+}$ shimming), channel‐wise relative $B_{1}^{+}$ maps were obtained^41^ with the following parameters: GRE, resolution: 1.5 × 1.5 × 6 mm^3^; TE/TR = 3.1/1510.4 ms; nominal FA = 15°; BW = 501 Hz/px; TA = 15 s. $B_{1}^{+}$ shimming was subsequently applied to locally maximize the $B_{1}^{+}$ efficiency (ƞ), which maximizes the $B_{1}^{+}$ magnitude per input power within a given ROI (ROI_shim_). This process was performed using a stand‐alone computer and custom‐built MatLab R2019b (MathWorks, Natick, MA) software.^39^ Because maximizing ƞ across the entire liver is not feasible at 7 T, the shim was optimized in an ROI_shim_ of approximately 30 mm diameter (Figure 2B).

Second‐order B_0_ shimming and subsequent B_0_ mapping (parameters: GRE, resolution: 1.5 × 1.5 × 6 mm^3^; TE/TR = 2.04/100 ms; nominal FA = 12°; BW = 781 Hz/px; TA = 34 s) were acquired using the corresponding $B_{1}^{+}$ shimming setting. If required, additional manual frequency adjustment was applied in ROI_*shim*_ by correcting the mean frequency shift within this ROI from the B_0_ mapping scan.

Two CEST images with two different mean nominal saturation amplitudes B_1_ = 0.6 and 0.9 μT (Gaussian‐shaped pulses, pulse duration 15 ms, duty cycle = 80%; number of RF pulses = 130 [saturation time t_sat_ = 2.43 s], recovery time t_rec_ = 3 s^42^) were acquired with a 2D GRE sequence^34^ (parameters: resolution: 1.5 × 1.5 × 6 mm^3^; TE/TR = 2.04/4.2 ms; nominal readout FA = 10°; BW = 1220 Hz/px). Z‐spectra were acquired for 73 frequency offsets Δω in unequal steps from −150 to +150 ppm relative to the water frequency. An additional image obtained with Δω = +300 ppm and t_rec_ = 12 s was acquired for normalization. Total TA for each CEST B_1_ value was 8 min 14 s. The acquisition duration (t_acq_) of each single offset (i.e., t_sat_ + t_acq_ + t_rec_ = 6.73 s) was synchronized with the natural respiratory cycle. Participants were instructed and trained to align their breathing pattern with the pulse sequence according to the sequence's sound: Whereas unrestricted breathing was allowed during saturation, the participants were instructed to hold their breath on exhale during image readout (Figure S3).

The $B_{1}^{+}$ distribution was mapped using an absolute, accurate, MR fingerprinting–based $B_{1}^{+}$ mapping technique^43^ (parameters: resolution: 1.5 × 1.5 × 6 mm^3^; TE/TR = 2.35/4.7 ms; nominal FA_nom_ = 40°; BW = 590 Hz/px; TA = 40 s). Relative $B_{1}^{+}$ values (*r*B_1_) were defined as the ratio of the obtained *actual* FA to *nominal* FA (= 40°):

(1) $$rB_{1}=\frac{B_{1,\mathrm{act}}}{B_{1,\mathrm{nom}}}=\frac{{\mathrm{FA}}_{\mathrm{act}}}{{\mathrm{FA}}_{\mathrm{nom}}}.$$

### 2.2 Participants

Five healthy volunteers (25 ± 3 years old; female/male: 3/2) with normal weight body mass index (20.3 ± 1.2) and without known fatty liver disease were recruited after approval of the study by the ethics committee of the Medical Faculty Heidelberg, Germany (S‐154/2014). All volunteers were provided with a full description of the study and gave signed informed consent in accordance with the institutional guidelines. To demonstrate the necessity and effectiveness of $B_{1}^{+}$ shimming for the CEST acquisition, the MRI protocol for one volunteer (subject 1) was extended with two additional CEST and $B_{1}^{+}$ maps acquired with (i) the default, non‐optimized transmit phase setting (identical phase across all transmitter channels), and (ii) the CP‐like transmission mode (45° phase shift), resulting in a total scan time of 40 min.

### 2.3 Calculation and processing of CEST image contrast

All image processing was conducted within MatLab R2019b (MathWorks). An adaptive coil combination algorithm was utilized for image reconstruction and phasing of the acquired MR raw data.^44^, ^45^ Reconstructed and B_0_‐corrected CEST data *M*_z_(Δω) was pixel‐wise normalized by one fully relaxed image *M*_0_ obtained with off‐resonance irradiation at Δω = +300 ppm to yield the Z‐spectrum. Regions with vessels and visible artifact were masked out (c.f. Figure S4). A five‐pool Lorentzian fit analysis (comprising the following parameters: direct water saturation (DWS) at Δω_DWS_ = 0 ppm, amide at Δω_amide_ = 3.5 ppm, guanidino at Δω_gua_ = 2.0 ppm, rNOE at Δω_rNOE_ = −3.5 ppm, and ssMT at Δω_ssMT_ = −2.7 ppm), was used for voxel‐wise Z‐spectrum fitting. The Fitting error (FE) was calculated for each signal as SD of the fitting residual divided by the amplitude of the fitted peak.^46^ All voxels with Fitting error > 25% were excluded. The isolated rNOE, amide, and guanidino CEST contrasts were calculated using the inverse contrast metric, that is, the MTR of the exchange‐dependent relaxation rate in the rotating frame (MTR_Rex_):

(2) $${\mathrm{MTR}}_{R_{\mathrm{ex}}}=\frac{R_{\mathrm{ex}}}{R_{1w}}=\frac{1}{Z_{\mathrm{lab}}}-\frac{1}{Z_{\mathrm{ref}}}.$$

Finally, a two‐point contrast B_1_ correction (B_1_ = 0.6 and 0.9 μT, reconstructed B_1_ = 0.8 μT) was applied as described by Windschuh et al.,^42^ resulting in the B_1_‐corrected amide, guanidino, and rNOE MTR_Rex_ maps. The fat fraction (FF) was estimated from the Z‐spectra as described by Zimmermann et al.^47^

To assess the reproducibility of the MTR_Rex_ values, the between‐subject coefficient of variation (COV) was calculated for each signal (amide, rNOE, and guanidino) within individual ROIs, defined as the ratio of the SD to the absolute mean.^48^

## 3 RESULTS

Structural images (CEST *M*_0_ at Δω = +300 ppm) (Figure 2A) obtained with three different phase settings in subject 1 demonstrate varying signal distributions throughout the liver. A substantial signal dropout in the center of the image occurred with default shimming, whereas both the CP‐like phase setting and $B_{1}^{+}$ efficiency shimming yielded higher $B_{1}^{+}$ magnitudes within the ROI in the liver region (c.f. Figure 2A). Although the improvement in $B_{1}^{+}$ magnitude from CP mode excitation to $B_{1}^{+}$ efficiency shimming is less apparent in the structural images, it is clearly visible in the *r*B_1_ maps (Figure 2B). Within the ROI_shim_, *r*B_1_ achieves a maximum of 0.95 ± 0.02 after $B_{1}^{+}$ shimming compared to 0.60 ± 0.03 with CP and 0.51 ± 0.04 with default phase settings.

The importance of achieving sufficiently high $B_{1}^{+}$ values for reliable CEST assessment is evident in representative Z‐spectra (Figure 2C) from a single voxel within ROI_shim_ with B_1,nom_ = 0.9 μT and *r*B_1_ = 0.49, 0.59, and 0.95 for default, CP phase settings, and $B_{1}^{+}$ efficiency shimming, respectively. Corresponding to the *r*B_1_ values, it becomes apparent in Figure 2C that the Z‐spectrum quality improves from the default to CP phase setting, with an even more obvious improvement when applying the efficiency shim. The high‐quality Z‐spectra obtained with the efficiency shim enabled accurate fitting using a five‐pool Lorentzian model, allowing for the spectral separation of individual CEST resonances as illustrated in Figure 2D.

**FIGURE 3:**
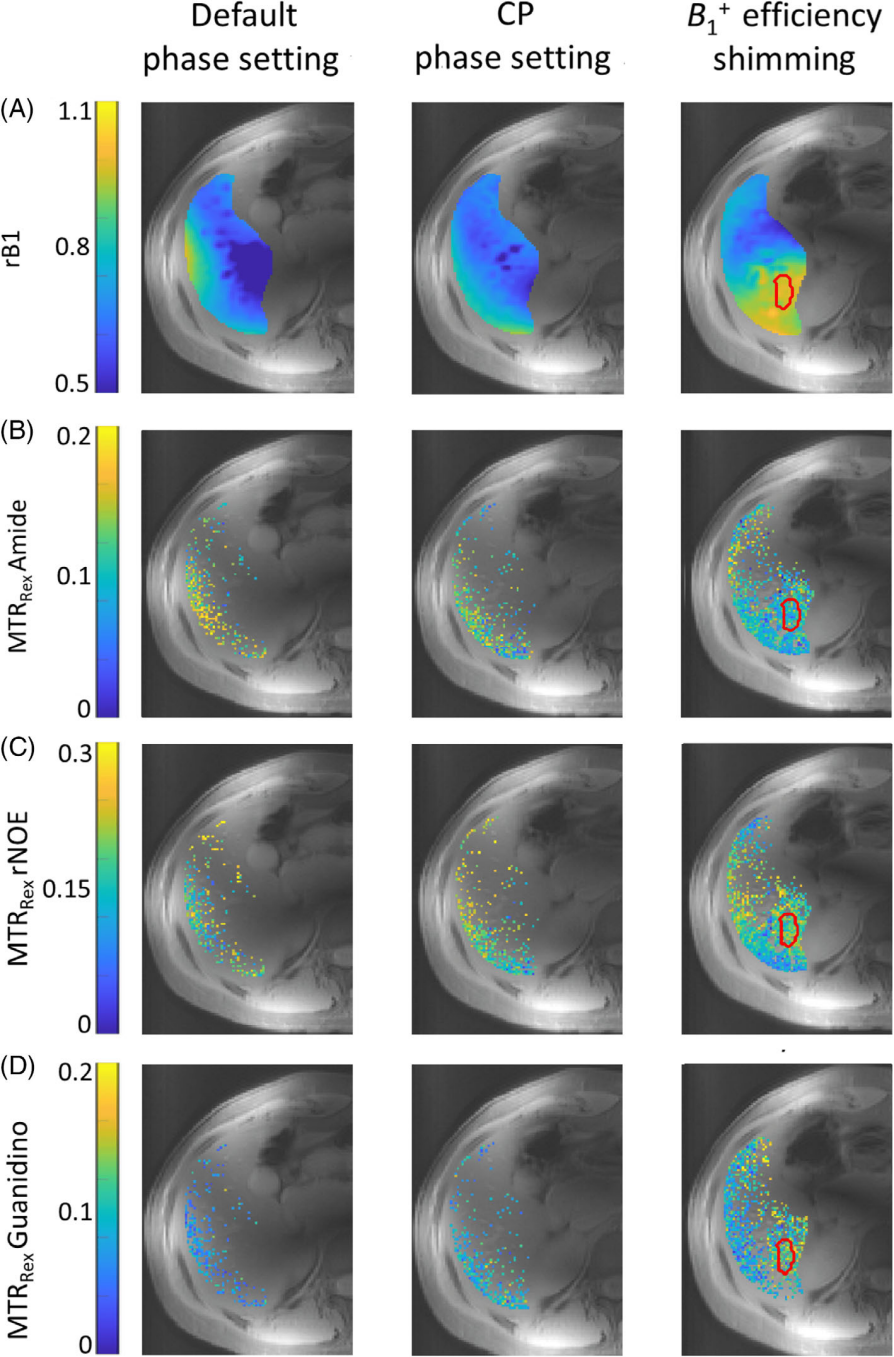
*r*B_1_ maps (A) and amide (B), rNOE (C), guanidino (D) MTR_Rex_ maps (reconstructed to nominal B_1_ = 0.8 μT) acquired with three different phase settings: default (left column), CP (middle column), and the optimized static phase setting ($B_{1}^{+}$ efficiency shimming, right column). The ROI (ROI_shim_) used for the $B_{1}^{+}$ shimming procedure is indicated by the red line. Regions with vessels and visible artifacts were masked out. Voxels with fitting error >25% were excluded. It is visible that the number of voxels with reliable MTR_Rex_ data in ROI_shim_ and adjacent tissues increases substantially with $B_{1}^{+}$ efficiency shimming. Colorbar of *r*B_1_ maps differs from that on Figure 2. MTR_Rex_, magnetization transfer ratio.

Extraction of the individual CEST resonances enabled the quantification of corresponding MTR_Rex_ values. MTR_Rex_ maps for amide, guanidino, and rNOE in subject 1, along with the corresponding *r*B_1_ maps, are shown in Figure 3 for the three different phase settings. Data is shown within the liver contour, excluding regions with vessels and visible artifacts in the images. Additionally, voxels with artifacts in the Z‐spectrum, caused by motion and strong B_1_/B_0_ inhomogeneities, were excluded. CP and the default phase setting allow for reliable MTR_Rex_ quantification only in a confined region along the liver edge, near the coil elements, which corresponds to regions with higher *r*B_1_ values (Figure 3A). In contrast, with an efficiency shim, high *r*B_1_ values extend deeper into the liver tissue, enabling reliable MTR_Rex_ quantification within ROI_shim_ and adjacent tissues, with the exception of regions containing vessels.

**FIGURE 4:**
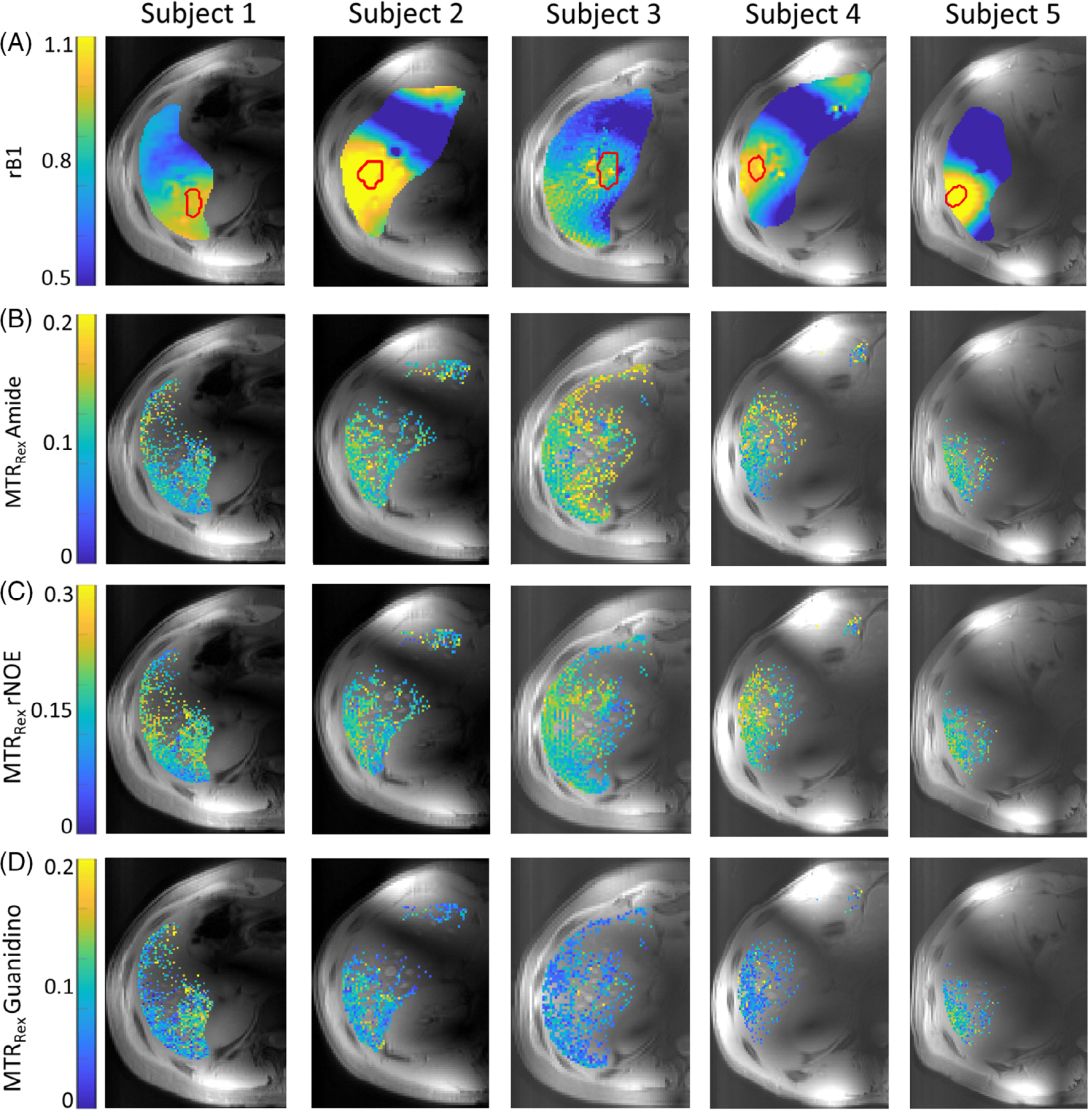
Results for all five subjects measured: *R*B_1_ maps (A) and amide (B), rNOE (C), guanidino (D) MTR_Rex_ maps (reconstructed to nominal B_1_ = 0.8 μT). The individual shimming ROIs (ROI_shim_) for each subject are indicated by the red line on the *r*B_1_ maps (A). Regions with vessels and visible artifacts were masked out. Voxels with fitting error >25% were excluded.

**FIGURE 5:**
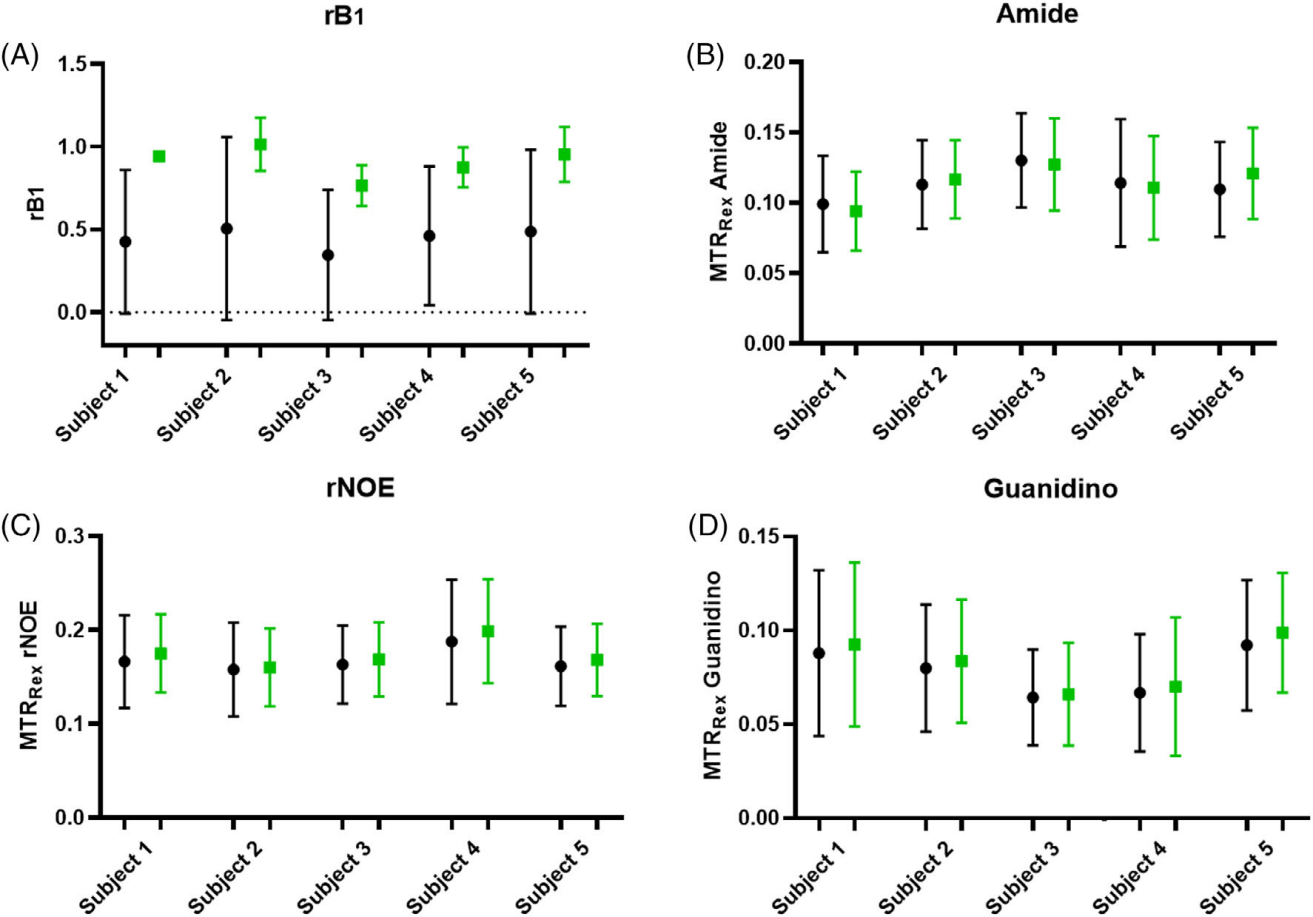
Mean values with SDs of *r*B_1_ values (A) and MTR_Rex_ contrasts (amide (B), rNOE (C), guanidino (D)) for all subjects. Corresponding values are taken from shimming ROIs (ROI_shim_, marked by red lines in Figure 4A) and are represented by green square markers, whereas values from all displayed voxels (Figure 4B–D) are represented by black circle markers.

MTR_Rex_ maps, reconstructed to a nominal B_1_ = 0.8 μT for all five measured subjects, along with the corresponding *r*B_1_ maps, are shown in Figure 4. Additionally, MTR_Rex_ maps obtained at nominal B_1_ of 0.6 μT and 0.9 μT are summarized in Figure S5. Within the ROI_shim_, reliable MTR_Rex_ values could be calculated for an average of 85% of voxels. For evaluation of the results, all shown (Figure 4B–D) voxels as well as voxels only within ROI_shim_ were used. Quantified results for MTR_Rex_ and *r*B_1_ values are summarized in Figure 5 using mean values with SDs. The mean MTR_Rex_ values and corresponding COVs across the group for all shown voxels are: 0.113 ± 0.009, 8.8% for amide; 0.167 ± 0.010, 6.3% for rNOE; and 0.079 ± 0.010, 12.9% for guanidino. For voxels within ROI_shim_: 0.112 ± 0.011 for amide, 0.169 ± 0.015 for rNOE, and 0.081 c0.012 for amine MTR_Rex_ values exhibit low variation between subjects, as reflected by low COVs. The average FF across all subjects was 1.48 ± 0.53%.

## 4 DISCUSSION

In the present study, to the best of our knowledge we have demonstrated for the first time the feasibility of acquiring and quantifying relaxation‐compensated CEST contrasts in the human liver at ultrahigh field (≥7 T). The application of static pTx effectively eliminates $B_{1}^{+}$ dropouts and allows for accurate CEST contrast quantification within the selected ROI_shim_. In addition, we could demonstrate that the recently proposed^43^ $B_{1}^{+}$ mapping technique is highly efficient for B_1_ correction of MTR_Rex_ contrast within the abdominal area.

In this study, we underline the importance of achieving sufficiently high *r*B_1_ values within a target ROI to enable accurate Z‐spectrum quantification and enhance the reliability of CEST imaging results. Specifically, for B_1_ correction to 0.8 μT, the required condition is *r*B_1_ × 0.9 μT >0.8 μT, which implies that *r*B_1_ should be greater than approximately 0.89. However, achieving optimal *r*B_1_ values in the desired ROI is substantially more challenging in 7 T abdominal imaging than in the brain, particularly in the liver.^49^

We demonstrated that this issue can be addressed locally very effectively using static pTx. Although both CP and default phase settings result in suboptimal and uneven $B_{1}^{+}$ distribution, limiting the regions where MTR_Rex_ values can be reliably quantified, $B_{1}^{+}$ shimming allowed improved *r*B_1_ values to be achieved in the target region (ROI_shim_) for each subject. It was shown that MTR_Rex_ values can be consistently calculated within and around ROI_shim_ if voxels affected by vessels and other artifacts are excluded. These findings are promising for the clinical application of CEST contrasts in the liver, regardless of the specific localization of pathology.

However, even with an optimized static pTx excitation, $B_{1}^{+}$ inhomogeneity persists, especially in large target volumes. Consequently, its effect on the CEST contrast needs to be mitigated through the B_1_ correction,^42^ which requires precise $B_{1}^{+}$ mapping. In this study, we employed an MR fingerprinting–based $B_{1}^{+}$ mapping technique.^43^ The radial acquisition combined with short acquisition time provides reliable $B_{1}^{+}$ maps even under free breathing, making the technique especially relevant for abdominal studies.

Our work shows that 7 T allows for the isolation of rNOE, guanidino, and amide CEST signals in the human liver, enabling the calculation of corresponding MTR_Rex_ values. These CEST effects are promising for clinical applications due to their correlation with biochemical tissue parameters, such as protein concentration,^50^, ^51^ intracellular pH,^29^, ^52^ and protein folding,^53^ as well as protein aggregation processes and interaction with lipid membranes.^54^ This makes it highly promising to apply the results of the current study to investigate pathological conditions in the liver. Liver CEST imaging, in particular, might be promising for assessing liver fibrosis,^18^ predicting the histologic grade of hepatocellular carcinoma^25^ and differentiating diseases such as focal liver lesions.^20^ Furthermore, liver CEST imaging shows potential as a liver cancer biomarker^55^ and has been utilized for differentiating benign and malignant liver tumors.^23^

Nevertheless, several limitations of our study should be acknowledged. First, the effective size of the ROI_shim_ is constrained. An efficient $B_{1}^{+}$ shim fails in most subjects if the ROI encompasses the entire liver. However, the size of the available ROI_shim_ is sufficient for accurate MTR_Rex_ quantification in a substantial portion of the liver, potentially covering the majority of liver tumors and pathological foci. Second, breathing synchronization in this study was achieved by aligning the subject's breathing with the gradient sounds, which is suboptimal and varies between individuals. Future implementation of more advanced respiratory motion correction techniques is necessary to enhance the accuracy and reliability of the results. Additionally, 7 T is associated with significant specific absorption rate constraints, which limit the flexibility of CEST sequence parameters. To mitigate this, we utilized a CEST sequence with an optimized and effective presaturation pulse train,^34^ allowing for reliable CEST imaging with the available sequence parameters.

This study does not apply any fat suppression or correction methods because the low FF ≈ 1.5% in the selected subjects has minimal impact on the Z‐spectra. However, due to the wide range of pathologies and age‐dependent physiological conditions that can lead to high FF in the liver, CEST fat correction methods remain a crucial area for further investigation.

Zhou et al.^22^ and Xu et al.^21^ identified the rNOE signal at −1 ppm from the glycogen aliphatic region in mouse liver at 11.4 T and in human liver at 3 T, respectively. However, in this study we did not include this signal in the approximation model due to its reduced intensity after fasting, as was previously shown,^19^, ^21^, ^22^ and the substantial overlap with DWS at 0 ppm, making it highly challenging to reliably separate the −1 ppm rNOE peak.

Further work beyond this study is needed to investigate motion correction strategies for CEST imaging of the liver. The feasibility of full liver volume CEST imaging and 3D acquisitions at 7 T also requires additional evaluation. Moreover, effective fat correction methods, which have not been applied in this work, should be explored to improve the accuracy and reliability of CEST measurements.

## 5 CONCLUSION

This study demonstrates that relaxation‐compensated CEST imaging can be performed reliably and reproducibly using $B_{1}^{+}$ shimming in combination with accurate $B_{1}^{+}$ mapping at 7 T. Future studies involving larger and more diverse samples of healthy volunteers and patients are needed to further validate the findings of this study. CEST MRI of the liver holds potential for providing complementary biochemical information on liver pathologies and warrants further investigation.

## Supporting information


**Figure S1.** (A–D) Body‐size (length = 500 mm, height = 240 mm, width = 350 mm) torso phantom with an off‐center tubular cut‐out (82 mm inner dia.) in the head‐foot direction, and a smaller, cylindrical phantom (diameter = 72 mm, length = 200 mm) inserted into the tubular cut‐out. (E) Two tubes (50 mL) were placed inside inner cylindrical phantom and contained Bovine Serum Albumin (BSA) water solutions with 7% and 10% concentrations and pH ˜7.
**Figure S2.** (A) Z spectra with following approximatin in representative voxels for 10% and 7% BSA solutions. (B) Phantom MTR_Rex_ maps for amide, guanidino and rNOE. (C) Mean with standard deviations for MTR_Rex_ of amide, guanidino and rNOE for 10% and 7% BSA solutions correspondingly.
**Figure S3.** Scheme of the breathing pattern applied for CEST acquisition. Participants were instructed to synchronize their breathing with the sequence's sound: they were allowed unrestricted breathing during the saturation phase but asked to hold their breath on exhale during image readout.
**Figure S4.** Vessels and visible artifacts masks for all subjects, Masked regions were excluded from further data processing.
**Figure S5.** Amide, rNOE and guanidino MTR_Rex_ maps for all five subjects, measured with nominal B_1_ = 0.6 and 0.9 μT correspondingly.
**Figure S6.** Representative Z‐spectra from liver with low fat fraction (FF) (approximately 1%) of subject 1 (A) and liver spectrum with high FF (approximately 10%) of one additional subject (Subject *) (B). Fat artifacts in Z‐spectrum are indicated in red (B). The FF was estimated from the Z‐spectrum.
