# Supplementary material for: Human liver CEST imaging at 7 T: Impact of B1+ shimming
Source: Magn Reson Med. 2025 May 24;94(4):1604–15. doi: 10.1002/mrm.30557 (PMC12309884; doi:10.1002/mrm.30557)
Supplement: Supplementary file 1 — Figure S1. (A–D) Body‐size (length = 500 mm, height = 240 mm, width = 350 mm) torso phantom with an off‐center tubular cut‐out (82 mm inner dia.) in the head‐foot direction, and a smaller, cylindrical phantom (diameter = 72 mm, length = 200 mm) inserted into the tubular cut‐out. (E) Two tubes (50 mL) were placed inside inner cylindrical phantom and contained Bovine Serum Albumin (BSA) water solutions with 7% and 10% concentrations and pH ˜7. Figure S2. (A) Z spectra with following approximatin in representative voxels for 10% and 7% BSA solutions. (B) Phantom MTRRex maps for amide, guanidino and rNOE. (C) Mean with standard deviations for MTRRex of amide, guanidino and rNOE for 10% and 7% BSA solutions correspondingly. Figure S3. Scheme of the breathing pattern applied for CEST acquisition. Participants were instructed to synchronize their breathing with the sequence's sound: they were allowed unrestricted breathing during the saturation phase but asked to hold their breath on exhale during image readout. Figure S4. Vessels and visible artifacts masks for all subjects, Masked regions were excluded from further data processing. Figure S5. Amide, rNOE and guanidino MTRRex maps for all five subjects, measured with nominal B1 = 0.6 and 0.9 μT correspondingly. Figure S6. Representative Z‐spectra from liver with low fat fraction (FF) (approximately 1%) of subject 1 (A) and liver spectrum with high FF (approximately 10%) of one additional subject (Subject *) (B). Fat artifacts in Z‐spectrum are indicated in red (B). The FF was estimated from the Z‐spectrum. [file MRM-94-1604-s001.docx]

**Supporting Information**

**Phantom study**

For phantom measurements, a body-size (length = 500 mm, height = 240 mm, width = 350 mm, volume = 35 l) PMMA torso phantom (Fig. S1a-b) was used. The phantom includes an off-center tubular cut-out (82 mm inner dia.) in the head-foot direction, and a smaller, cylindrical phantom (diameter = 72 mm, length = 200 mm) inserted into the tubular cut-out (Supporting Information Figure S1c-d). The volume of the phantom’s main body and inner cylindrical phantom were filled with a 25 mmol/l NaCl water solution. Two tubes (50ml) were placed inside the inner cylindrical phantom (Supporting Information Figure S1e) and contained Bovines Serumalbumin (BSA) water solutions with 7% and 10% concentrations and pH ~7.

The in-vitro scanning protocol, imaging system, and RF coil were identical to those used for in-vivo measurements. An efficient B_1_^+^ shim was calculated within the ROI of the inner cylindrical phantom and subsequently applied, which mitigated B_1_^+^​ inhomogeneities and prevented signal dropouts. The resulting Z-spectra (Supporting Information Figure S2a) exhibited high SNR and clear spectral separation, enabling the calculation of MTR_Rex_​ maps for amide, rNOE, and guanidino signals (Supporting Information Figure S2b). The measured MTR_Rex_​ values (Supporting Information Figure S2c) demonstrated a strong correlation with BSA concentration, as expected, confirming the reliability and quantitative validity of the data.


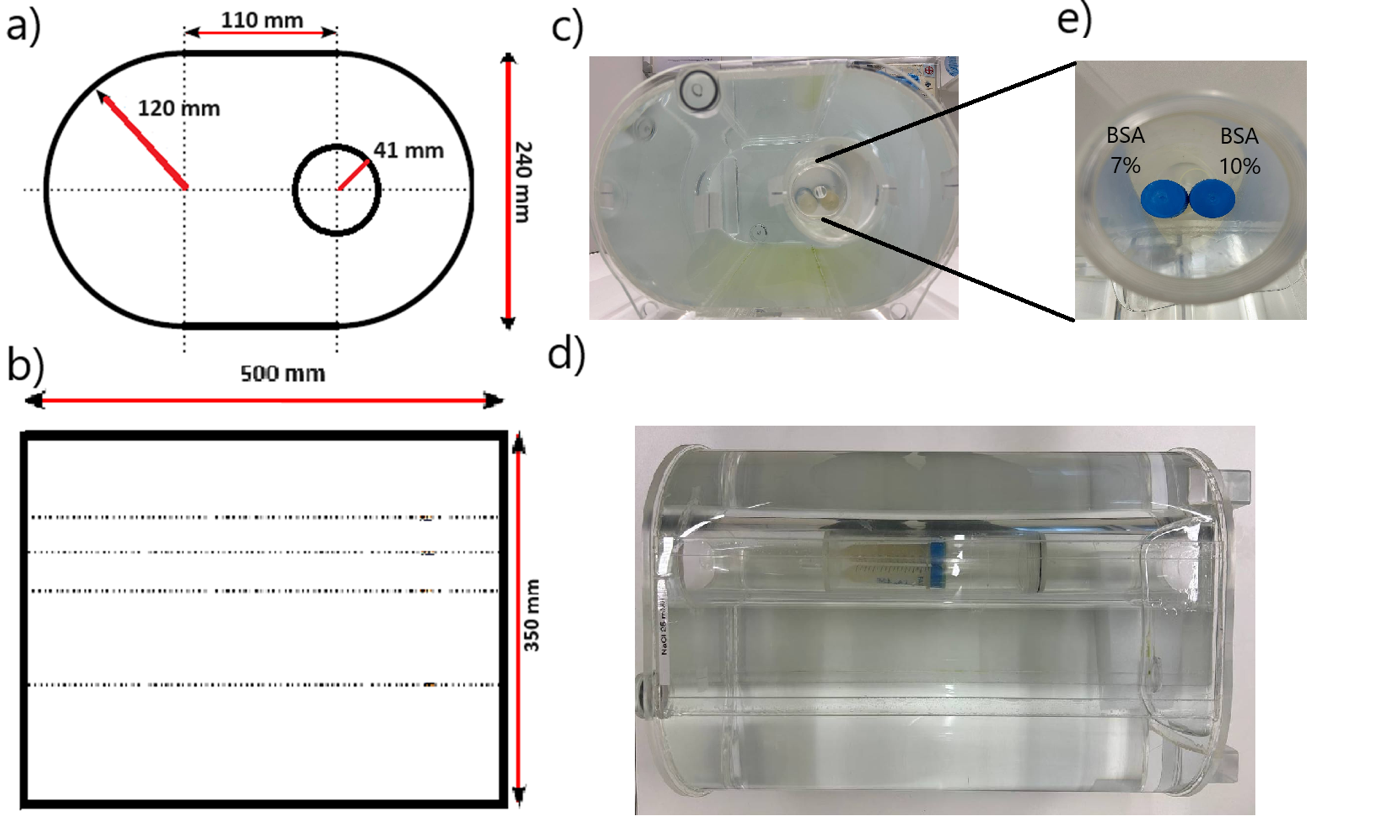


**Supporting Information Figure S1**. a-d. Body-size (length = 500 mm, height = 240 mm, width = 350 mm) torso phantom with an off-center tubular cut-out (82 mm inner dia.) in the head-foot direction, and a smaller, cylindrical phantom (diameter = 72 mm, length = 200 mm) inserted into the tubular cut-out. e) Two tubes (50ml) were placed inside inner cylindrical phantom and contained Bovines Serumalbumin (BSA) water solutions with 7% and 10% concentrations and pH ~7.


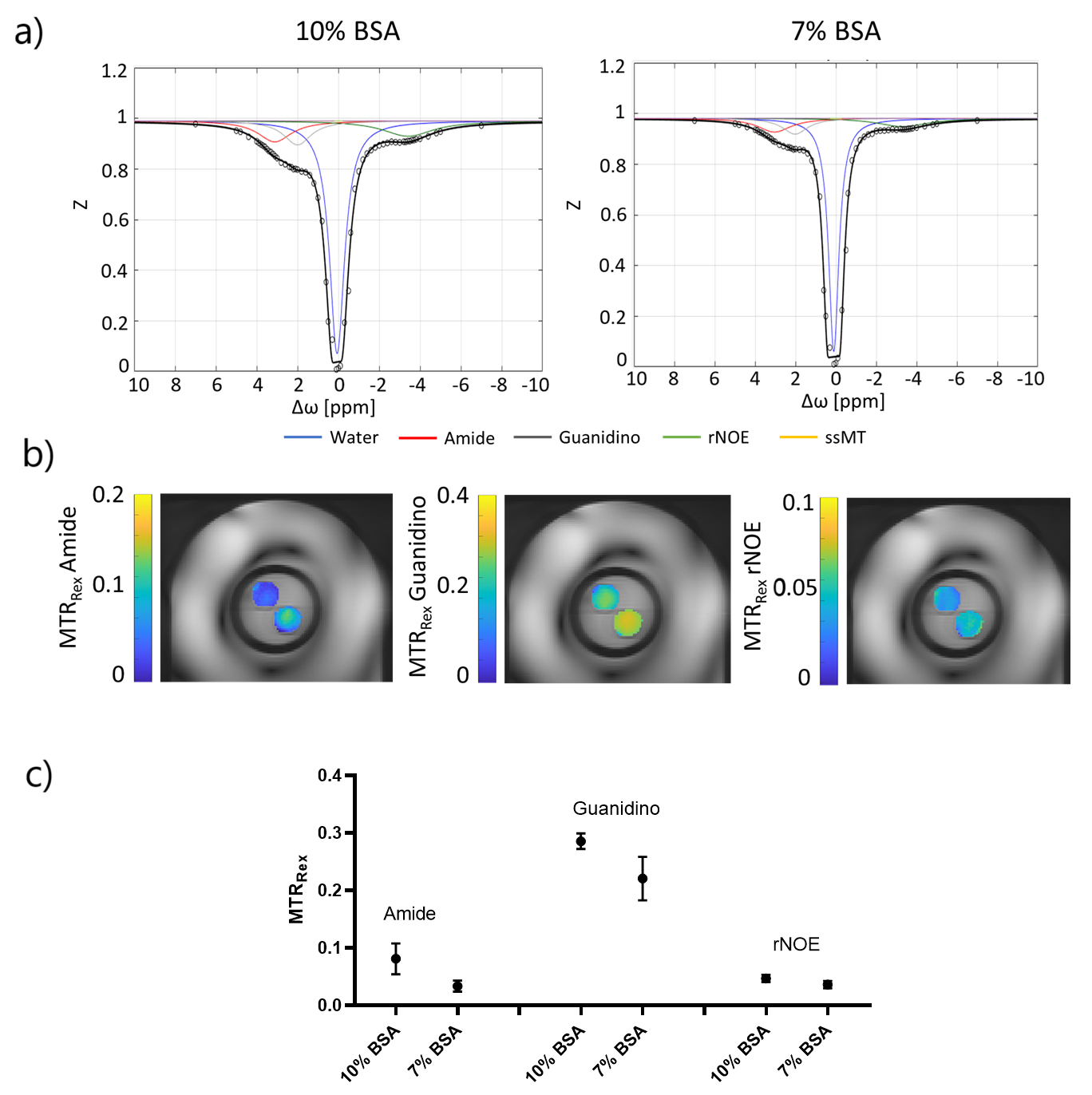


**Supporting Information Figure S2**. a) Z spectra with following approximation in representative voxels for 10% and 7% BSA solutions. b) Phantom MTR_Rex_ maps for amide, guanidino and rNOE. c) Mean with standard deviations for MTR_Rex_ of amide, guanidino and rNOE for 10% and 7% BSA solutions correspondingly.


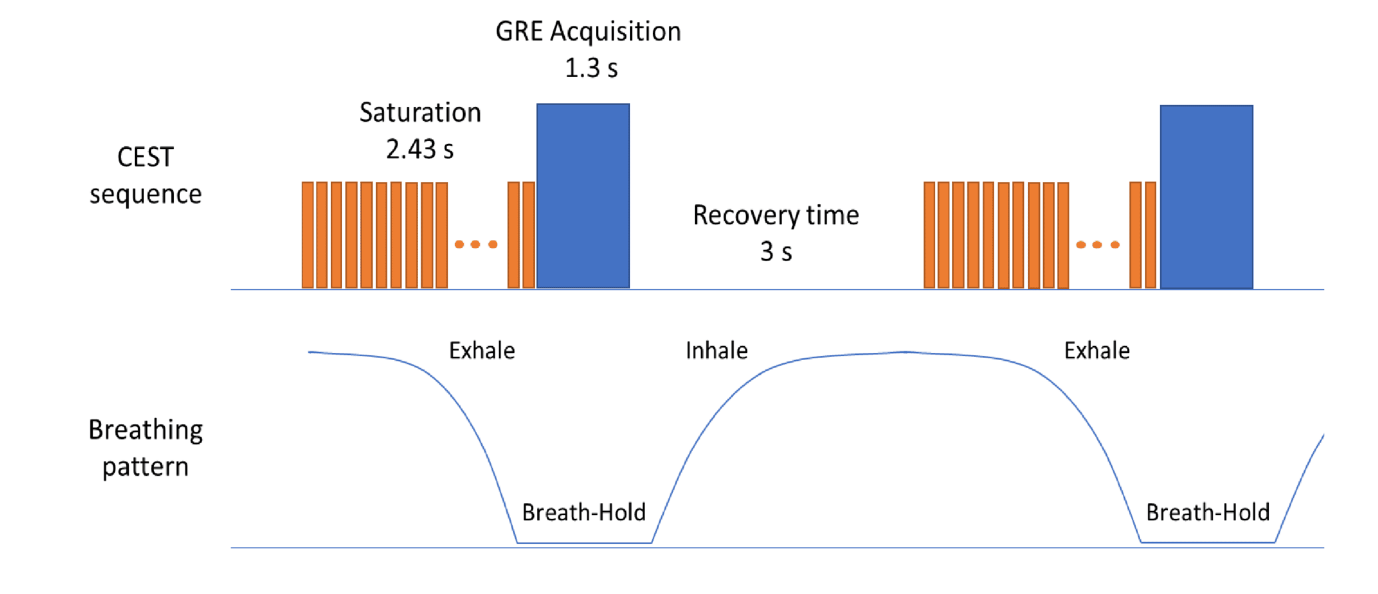


**Supporting Information Figure S3**. Scheme of the breathing pattern applied for CEST acquisition. Participants were instructed to synchronize their breathing with the sequence’s sound: they were allowed unrestricted breathing during the saturation phase but asked to hold their breath on exhale during image readout.


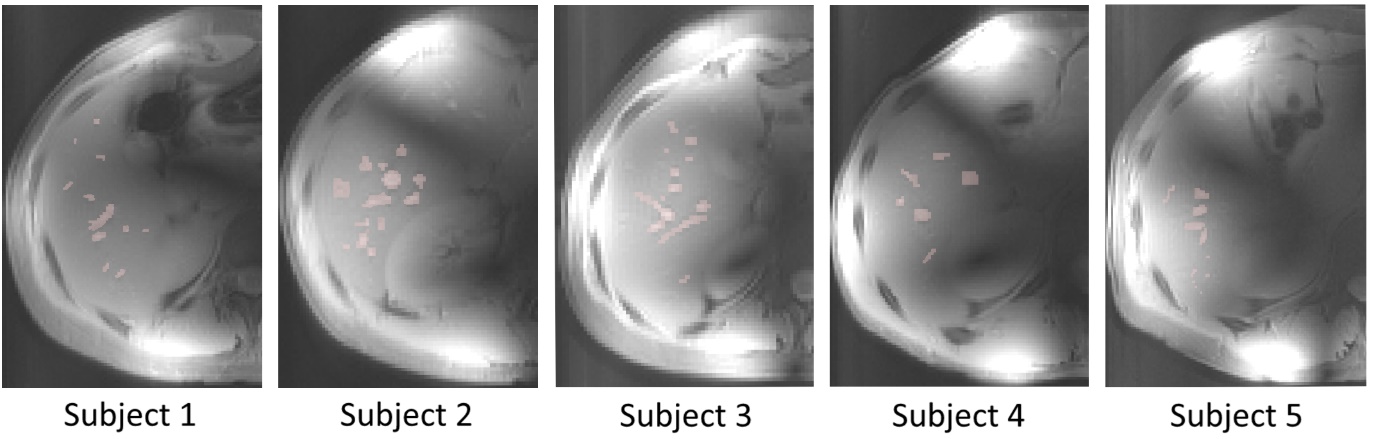


**Supporting Information Figure S4.** Vessels and visible artefacts masks for all subjects, Masked regions were excluded from further data processing.


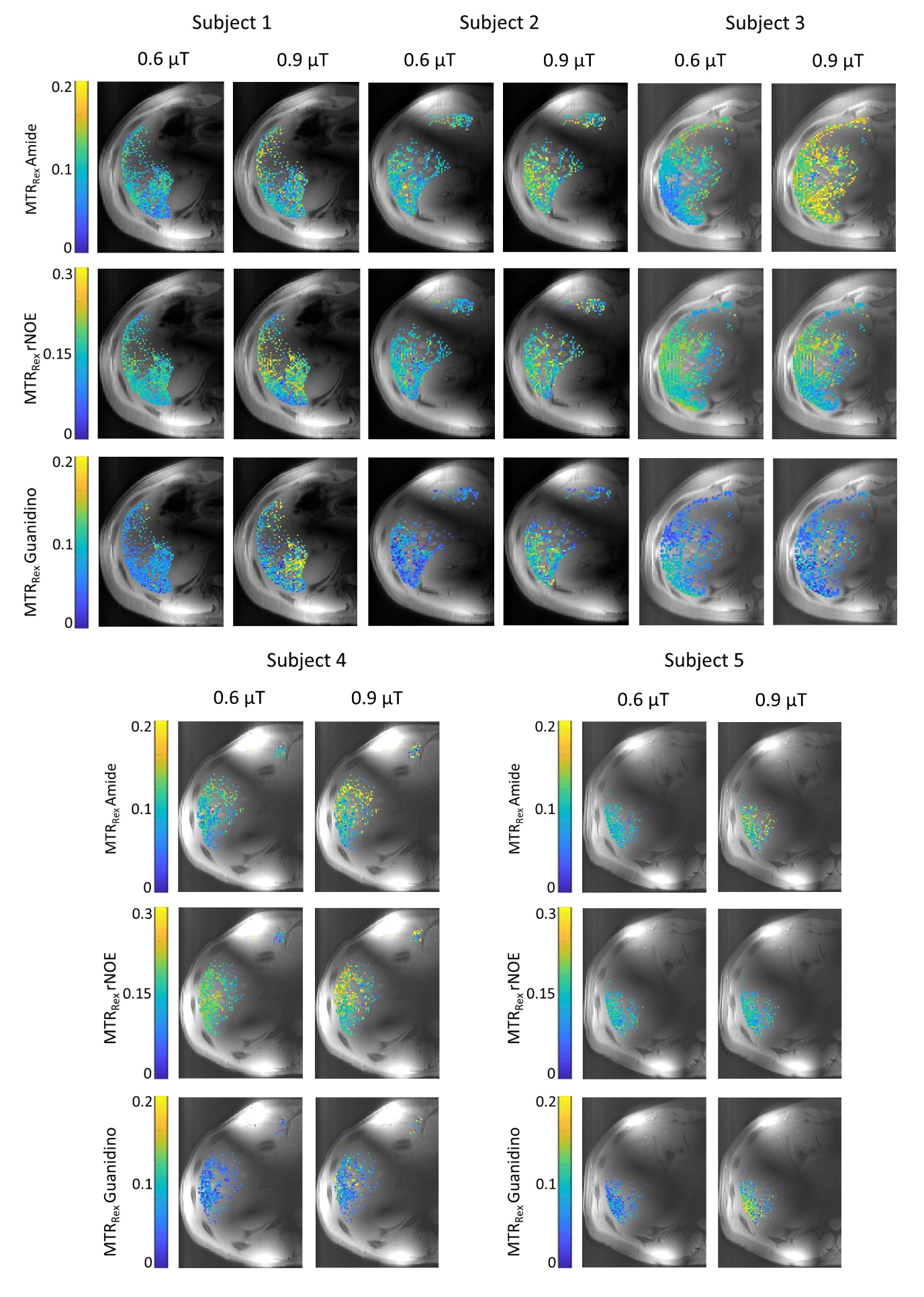


**Supporting Information Figure S5**. Amide, rNOE and guanidino MTR_Rex_ maps for all five subjects, measured with nominal B_1_ = 0.6 and 0.9 μT correspondingly.


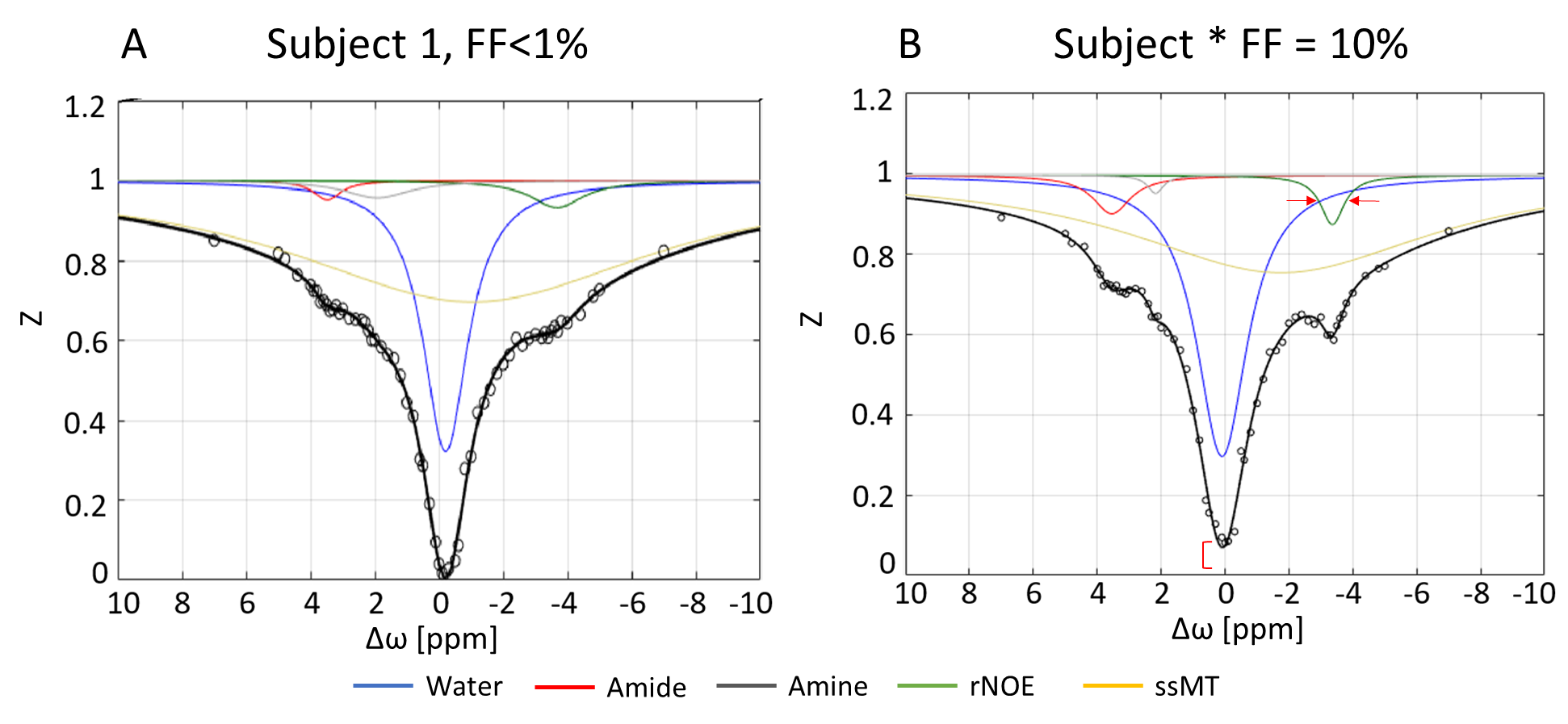


**Supporting Information Figure S6**. Representative Z-spectra from liver with low fat fraction (FF) (approximately 1%) of subject 1 (A) and liver spectrum with high FF (approximately 10%) of one additional subject (Subject *) (B). Fat artefacts in Z-spectrum are indicated in red (B). The FF was estimated from the Z-spectrum.
